# The neurotoxic secreted phospholipase A_2_ from the *Vipera a*. *ammodytes* venom targets cytochrome c oxidase in neuronal mitochondria

**DOI:** 10.1038/s41598-018-36461-6

**Published:** 2019-01-22

**Authors:** Jernej Šribar, Lidija Kovačič, Jernej Oberčkal, Adrijan Ivanušec, Toni Petan, Jay W. Fox, Igor Križaj

**Affiliations:** 1 Department of Molecular and Biomedical Sciences, Jožef Stefan Institute, Jamova 39, 1000 Ljubljana, Slovenia; 2 Faculty of Medicine, University of Ljubljana, Vrazov trg 2, 1000 Ljubljana, Slovenia; 3 Department of Microbiology, Immunology and Cancer Biology, University of Virginia School of Medicine, Charlottesville, Virginia 22908 USA

## Abstract

The β-neurotoxic secreted phospholipases A_2_ (sPLA_2_s) block neuro-muscular transmission by poisoning nerve terminals. Damage inflicted by such sPLA_2_s (β-ntx) on neuronal mitochondria is characteristic, very similar to that induced by structurally homologous endogenous group IIA sPLA_2_ when its activity is elevated, as, for example, in the early phase of Alzheimer’s disease. Using ammodytoxin (Atx), the β-ntx from the venom of the nose-horned viper (*Vipera a*. *ammodytes*), the sPLA_2_ receptor R25 has been detected in neuronal mitochondria. This receptor has been purified from porcine cerebral cortex mitochondria by a new Atx-affinity-based chromatographic procedure. Mass spectrometry analysis revealed R25 to be the subunit II of cytochrome c oxidase (CCOX), an essential constituent of the respiratory chain complex. CCOX was confirmed as being the first intracellular membrane receptor for sPLA_2_ by alternative Atx-affinity-labellings of purified CCOX, supported also by the encounter of Atx and CCOX in PC12 cells. This discovery suggests the explanation of the mechanism by which β-ntx hinders production of ATP in poisoned nerve endings. It also provides a new insight into the potential function and dysfunction of endogenous GIIA sPLA_2_ in mitochondria.

## Introduction

Understanding the mechanism of the presynaptic neurotoxicity (β-neurotoxicity) exerted by phospholipases A_2_ (sPLA_2_s) secreted by some Elapidae and Viperidae snake venoms has presented a major challenge for several decades. Structurally, these toxins belong mostly to group I or II (GI or GII) sPLA_2_s^1^. The former are orthologues of the mammalian “pancreatic” GIB sPLA_2_ and the latter of the mammalian “inflammatory” GIIA sPLA_2_. In nature, these toxins, also termed β-neurotoxins (β-ntxs), target specifically the presynaptic membrane (PM) of motoneurons. Poisoning of the neuro-muscular junction by a β-ntx results in flaccid paralysis, characterized by blockade of the exocytosis of the neurotransmitter-filled synaptic vesicles (SVs), reduction of the number of SVs in the nerve ending, swelling and degeneration of mitochondria accompanied by release of alarmins and, finally, by destruction of the nerve terminal^2^. At the molecular level, β-ntxs initially bind, selectively, to a variety of, still unidentified, receptors in the axolemma. It is generally accepted that the phospholipase activity of β-ntxs is a very important factor, though not sufficient, for the full expression of neurotoxicity. Hydrolysis of the membrane phospholipids changes the transitivity, curvature, fluidity and fusogenicity of the membrane. Experiments on yeast *Saccharomyces cerevisiae*, the simplest eukaryotic cell, have suggested that enzymic activity at the appropriate location inside the cell is a basis for β-neurotoxicity^3^. Supporting this, a GIIA β-ntx - ammodytoxin (Atx) from the venom of the nose-horned viper (*Vipera a*. *ammodytes*) - was shown to be able to cross the PM of the mouse motor nerve terminal, the natural target tissue for the toxin, to reach the cytosol and mitochondria^4^. Cell internalization of β-ntxs has been shown using neuronal cell lines in culture. Atx added to the medium containing intact NSC-34 cells rapidly bound two cytosolic proteins in these cells, calmodulin (CaM) and 14-3-3 protein^5^. In primary cultures of spinal cord motor neurons and cerebellar granule neurons, GI β-ntxs, notexin, β-bungarotoxin and taipoxin targeted mitochondria selectively^6^. The β-ntxs induced the opening of mitochondrial permeability transition pores, uncoupled the mitochondrial transmembrane potential (Δ*Ψ*_m_) and inhibited the production of ATP. Supported by the discovery of a high-affinity membrane receptor for Atx in mitochondria of the porcine cerebral cortex, R25^7,8^, this organelle is clearly a very important target of β-neurotoxic sPLA_2_s in neuronal cells.

As is generally accepted, toxic venom proteins have evolved from physiological body proteins, usually those involved in key regulatory processes or bioactivity^9^. It is not surprising though that endogenous GIIA sPLA_2_, very similar in structure to Atx, has been discovered in mitochondria of mammalian cells^10^. Besides the confirmed involvement of this enzyme in neuritogenesis^11^, other very important pathophysiological functions of GIIA sPLA_2_ associated with mitochondria have been suggested^12,13^. Its association with the aetiology of some neurodegenerative diseases, for example Alzheimer’s disease^14–17^, is of the greatest relevance. A hallmark of induction of the latter is the elevated expression of GIIA sPLA_2_ in affected tissue, with concomitant dysfunction of neuronal mitochondria. The pathological effects of β-ntxs and GIIA on mitochondria are similar, so a description of the mode by which β-ntxs encounter and affect neuronal mitochondria on the molecular level would be expected to advance the study of the role of endogenous GIIA sPLA_2_ in these destructive diseases, leading, ultimately, to new diagnostic and therapeutic solutions. As an important breakthrough in this direction we report here the solution of the almost two-decade-old enigma of the molecular identity of R25, the mitochondrial receptor for Atx, the snake venom GIIA sPLA_2_.

## Results

### Purification of R25 from porcine cerebral cortex

**Figure 1:**
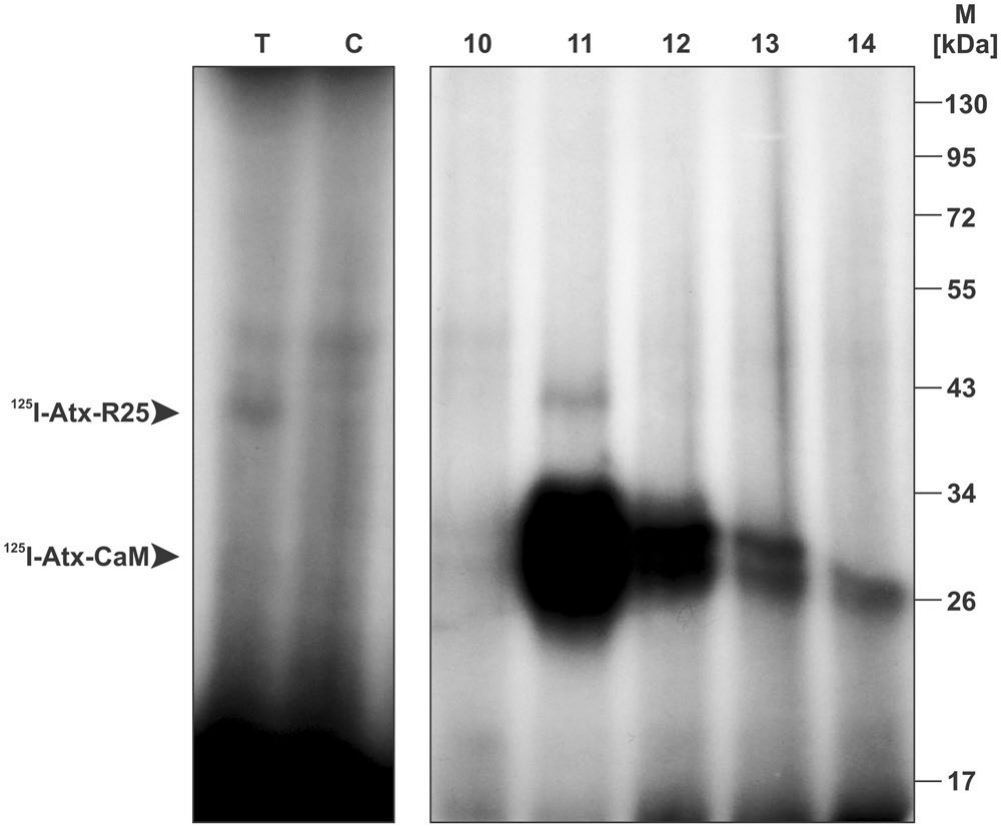
Affinity purification of R25. P2d membranes of porcine cerebral cortex were extracted using Triton X-100. Signals corresponding to specific Atx-binding proteins were identified by ^125^I-Atx/DSS affinity-labelling of the detergent extract followed by SDS-PAGE analysis and autoradiography of the gel (left panel). Affinity labelling performed in the absence of the native Atx is analysed in lane (T) while the one performed in the presence of 2 µM of native Atx is analysed in the lane (C). Using the same detection method, R25 has been located in fraction 11 from the Atx-CIM affinity chromatography (right panel). Details of affinity chromatography are described under Materials and methods. Arrowheads indicate positions of the specific adducts between ^125^I-Atx and CaM or R25, respectively. Full-length gel is shown. Different areas of the gel are divided by white space.

An appropriate source from which to isolate R25 was shown to be the mitochondria of the porcine cerebral cortex^8^. The starting material for a detergent extraction was therefore either the mitochondrial or the P2d subfraction of the demyelinised crude mitochondrial-synaptosomal fraction (P2) of this tissue. As established, the Atx-binding activity of R25 was unaffected by washing the membranes with 1 M NaCl^8^, so they were washed in this way to remove contaminating proteins as much as possible. The membrane proteins were extracted with 1.5% (m/v) Triton X-100. This was shown to be the lowest concentration of the detergent still providing optimal extraction of the receptor^8^. From this point on, all attempts to isolate R25 using standard purification techniques have so far failed due to inactivation of the receptor, reflected in the loss of its affinity for Atx. A very important factor enhancing inactivation of the receptor was the duration of the Atx-affinity-purification step. To speed up this stage of the purification procedure, CIM disks were employed, sustaining high flow rates of the mobile phase to prepare the Atx-affinity chromatography. Detergent extracts of the membranes were applied on the Atx-CIM disks packed in a chromatography cartridge attached to the FPLC apparatus. The disks were thoroughly washed and the retained proteins then eluted by the addition of EGTA to the mobile phase. R25 was traced in eluted fractions using ^125^I-Atx affinity-labelling (Fig. 1). The use of CIM disks therefore enabled the isolation of sufficient quantity of functional R25 protein.

### Molecular identification of R25

**Figure 2:**
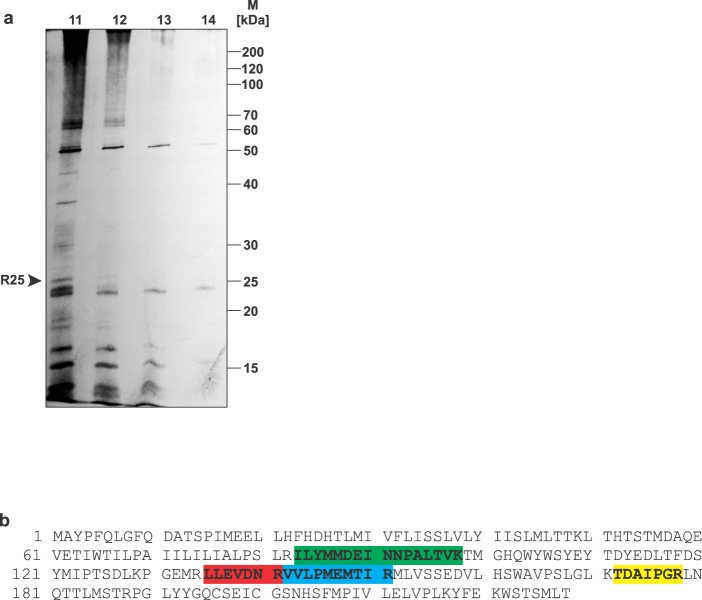
Structure of R25. (**a**) Porcine cerebral cortex P2d membranes were detergent-extracted and the extract chromatographed on an Atx-CIM affinity column. Proteins were eluted using 2 mM EGTA. Collected fractions were concentrated and analysed by SDS-PAGE. Proteins were visualized by silver-staining. As expected from the ^125^I-Atx/DSS affinity-labelling experiment (Fig. 1), the 25 kDa protein (R25) was concentrated in fraction 11. Full-length gel is shown. (**b**) The portion of gel containing R25 was excised, reduced, alkylated and submitted to in-gel trypsin digestion. The resulting peptides were extracted from the gel and analysed by LC-MS/MS. Four tryptic peptides from R25, shown in green, red, blue and yellow, were sequenced and identified as parts of the CCOX subunit II.

The R25-positive fraction from Atx-CIM-affinity chromatography was concentrated and analysed on SDS-PAGE. The receptor was located on the gel by silver staining (Fig. 2a). The piece of the gel with R25 was excised, reduced, alkylated and digested in-gel with trypsin. The resulting peptides were extracted from the polyacrylamide and their amino acid sequences determined using a liquid chromatography-tandem mass spectrometry (LC-MS/MS) system (Fig. 2b). Four peptides were sequenced. The identified parts of the R25 structure were found to be identical to those of 18% of the primary structure of subunit II of CCOX (CCOX-II), an essential component of the electron transport chain in mitochondria. CCOX-II is encoded by the mitochondrial gene *MT-CO2*. It is composed of 227 amino acids with a molecular mass of 25565 Da, which corresponds to the apparent molecular mass of R25.

### Atx binds to the purified CCOX

**Figure 3:**
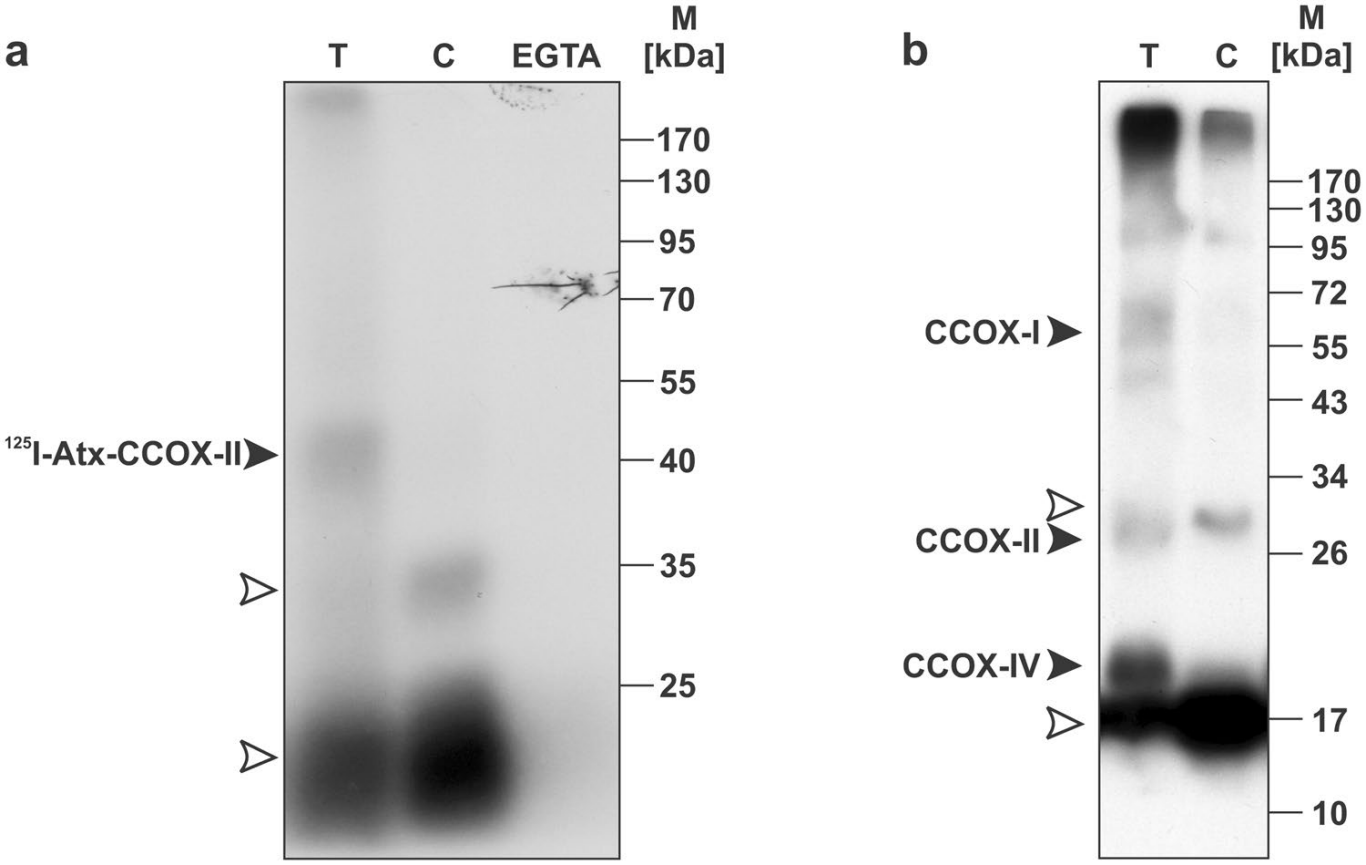
Atx binds specifically to purified CCOX. Purified bovine heart CCOX was incubated with ^125^I-Atx (**a**) or sulfo-SBED-Atx (**b**) in the absence (T) or presence of 200-fold (**a**) or 100-fold (**b**) molar excess of native Atx (C) over the labelled toxin, respectively. (**a**) Following the incubation, ^125^I-Atx was covalently cross-linked to CCOX, using DSS, the samples analysed by SDS-PAGE and the gel autoradiographed. Filled arrowhead indicates the position of the ^125^I-Atx–CCOX-II adduct. The experiment, in which Ca^2+^ ions were withdrawn by EGTA, revealed the Ca^2+^-dependence of the interaction between Atx and CCOX-II. Positions of ^125^I-Atx and its dimer are indicated by empty arrowheads. Full-length gel is shown. (**b**) After incubation with the sulfo-SBED-Atx, the samples were illuminated with UV light of 312 nm and analysed by SDS-PAGE. Proteins were Western-blotted from the gel to the PVDF membrane. Biotinylated proteins on the membrane were revealed using the SA-HRP system. Filled arrowheads indicate biotinylated subunits I, II and IV of CCOX. The monomer and dimer of the biotinylated Atx are indicated by empty arrowheads. Full-length blot is shown.

To confirm that the target of the neurotoxic sPLA_2_ in mitochondria is indeed CCOX, two, alternative, affinity-based labelling procedures were performed on a commercially available bovine heart CCOX. In the first, the radioactive derivative of Atx, ^125^I-Atx, and a cross-linker DSS were used while, in the second, the photo-reactive derivative of Atx, sulfo-SBED-Atx, was employed. As is evident from the affinity-labelling pattern (Fig. 3a), only one specific adduct, with an apparent molecular mass 39 kDa, resulted from the ^125^I-Atx/DSS labelling. The molecular mass of Atx is about 14 kDa, so that of the Atx-binding CCOX subunit in the complex was about 25 kDa, corresponding to the mass of subunit II. If the experiment was carried out in the absence of Ca^2+^, by the addition of EGTA, the specific adduct was not formed. The affinity-labelling pattern of CCOX with sulfo-SBED-Atx was more complex (Fig. 3b). As well as to subunit II, the biotin label was also transferred specifically from the Atx-derivative to subunits I and IV of CCOX with molecular masses 57 and 17.2 kDa, respectively. This is not surprising. The photo-reactive arylazido group of sulfo-SBED-Atx is less selective than the succinimido group of DSS and reacts with a wider spectrum of nucleophilic groups. CCOX is a large protein complex residing in the inner mitochondrial membrane. It is a dimer and each of the monomers is composed of 13 different subunits. The CCOX subunits I, II and IV are sterically similar to one another^18^ so, as expected, a less selective labelling procedure labelled the area around the Atx binding site more extensively.

### PC12 cells are a suitable model cell line on which to study the interaction of Atx with mitochondria

**Figure 4:**
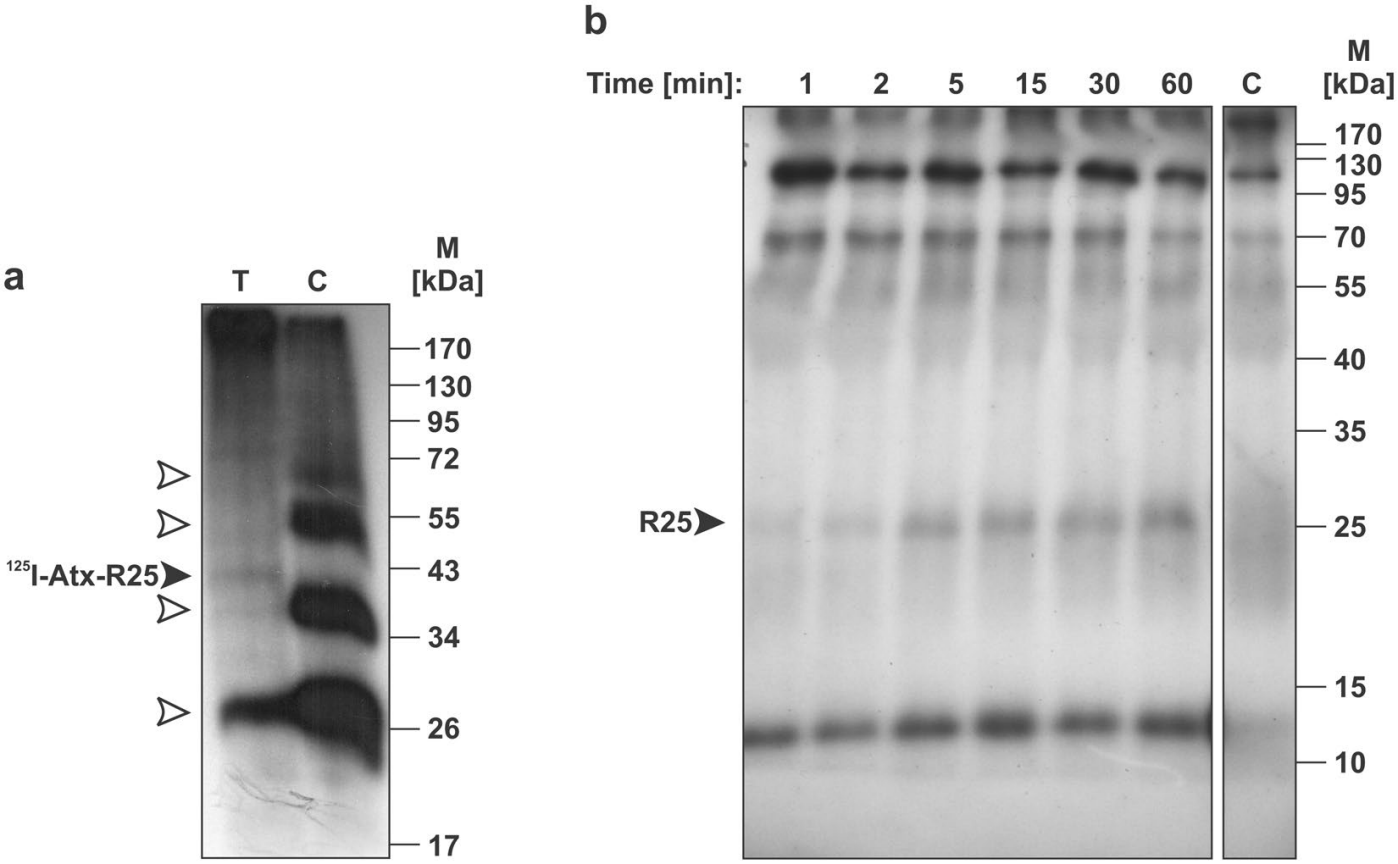
Atx also binds to 25 kDa specific receptor in PC12 cells. (**a**) Mitochondrial membranes of PC12 cells were incubated with ^125^I-Atx (T). In the control sample (C) 200-fold molar excess of the native Atx over the radiolabelled Atx was present during the incubation. Formed protein complexes were covalently cross-linked with DSS. Affinity-labelled membranes were dissolved and analysed by SDS-PAGE. The resulting gel was autoradiographed. The filled arrowhead indicates the specific adduct of ^125^I-Atx and a protein of 25 kDa (R25). The positions of ^125^I-Atx and its oligomers are indicated by empty arrowheads. Full-length gel is shown. (**b**) PC12 cells were incubated in the dark with the photoreactive sulfo-SBED-Atx for indicated periods of time. Each sample was exposed to UV light to trigger photo-cross-linking between the biotin-containing Atx-derivative and proteins in its close proximity. Cells were detergent-extracted and the biotinylated proteins isolated on avidin-beads. From the latter, just the fraction of biotinylated Atx-containing proteins was obtained by precipitation with anti-Atx antibodies. Samples were further analysed by SDS-PAGE. Proteins were transferred from the gel to the PVDF membrane and those biotinylated revealed by the SA-HRP detection system. The control sample (C) was processed without the addition of sulfo-SBED-Atx. Filled arrowhead is pointing at the specifically labelled band of about 25 kDa demonstrating that Atx is able to interact with R25 also in living PC12 cells. Full-length blot is shown. Different areas of the blot are divided by white space.

Rat adrenal pheochromocytoma cells, PC12, exhibit properties similar to those of neurons^19^. The fact that the sulfo-SBED-Atx derivative was able to enter the cytosol of PC12 cells rapidly qualified these cells as suitable for studying the pathway of the neurotoxic sPLA_2_ from the external space, through the plasma membrane and into the cytosol^20^. As shown in this work, PC12 cells harbour a specific receptor for Atx, R25, in their mitochondrial membranes. The same receptor was also detected in the mitochondria of porcine cerebral cortex. Importantly, this receptor was not only labelled specifically with the Atx probe (^125^I-Atx/DSS) on isolated P2d membranes (Fig. 4a), but was also tagged in an experiment employing sulfo-SBED-Atx on living PC12 cells (Fig. 4b). This means that the mechanism exists in PC12 cells, as in motoneurons, the natural target cells of β-ntxs, by which sPLA_2_ neurotoxin added to the cell media is able to reach mitochondria inside these cells. The use of PC12 cells as model cells can thus be extended to studying the interaction of the neurotoxic sPLA_2_s with mitochondria.

### Atx and CCOX-II colocalize in PC12 cells

**Figure 5:**
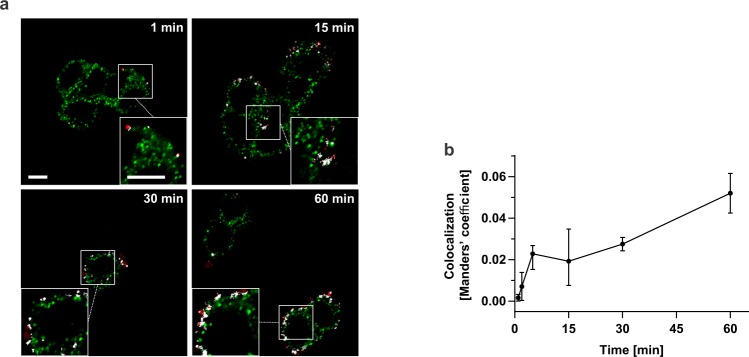
Atx and CCOX-II can encounter in living PC12 cells. (**a**) PC12 cells were incubated in the presence of 100 nM ^546^Alexa-tagged Atx (red signal) for the indicated periods of time. Cells were fixed, CCOX-II stained using the anti-CCOX-II antibodies (green signal) and then analysed under a confocal microscope. Co-localized green and red signals are shown in white. The magnified views of the selected areas of cells in insets are exhibiting the co-localization of signals even better. Experimental details are described in Materials and methods. Scale bars correspond to 5 µm. (**b**) Using stacks of acquired images, the extent of colocalization of Atx and CCOX-II in PC12 cells was calculated, expressed in terms of Manders’ coefficient and presented as a function of time of incubation of the cells with the toxin. Points represent the means and the bars represent the minimum and maximum values of the coefficient calculated from at least two sets of images for any given time point.

Following the demonstration that PC12 cells constitute a relevant model system on which to study the effects of neurotoxic sPLA_2_s on mitochondria, additional confirmation that CCOX-II is the target for Atx in neuronal mitochondria was sought by determining the extent of colocalization between Atx and CCOX-II in these cells. To this end, PC12 cells were incubated with ^546^Alexa-Atx (red) for different periods of time, fixed and labelled with anti-CCOX-II antibodies (green). The distribution of both red and green signals in the cells was determined using confocal laser microscopy by acquiring several optical sections for each sample (Fig. 5a). Stacks of images of each sample were quantified and the degree of colocalization of the red and the green signal determined using ZEISS ZEN software. Autofluorescence and background signals were eliminated by control experiments omitting the addition of either ^546^Alexa-Atx or ^488^Alexa-conjugated secondary antibodies, in order to determine the threshold signal for each channel, using ZEISS ZEN software. As revealed in the control experiment, the ^546^Alexa dye which was not covalently attached to the protein did not contribute to the red signal. The results are represented in terms of Manders’ coefficient (Fig. 5b). While low colocalization of signals for ^546^Alexa-Atx and CCOX-II was observed at incubation times up to 2 min, colocalization became considerable at incubation times longer than 5 min. These results thus additionally support the conclusion that R25, the mitochondrial receptor of Atx, and the subunit II of CCOX are the same protein. The results thus suggest that the *in vitro* established binding of Atx to CCOX is biologically relevant.

### Atx inhibits the enzymic activity of CCOX

**Figure 6:**
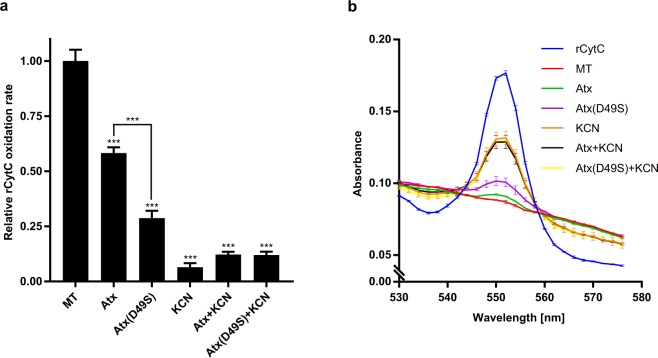
Atx inhibits the enzyme activity of CCOX. (**a**) Mitochondria were isolated from PC12 cells and incubated in either the absence (MT) or the presence of 1 µM Atx, 1 µM Atx(D49S), 0.5 mM KCN, 1 µM Atx with 0.5 mM KCN or 1 µM Atx(D49S) with 0.5 mM KCN. After the addition of the CCOX substrate, rCytC, the change in absorbance at 550 nm (A_550_) was measured. The relative rCytC oxidation rates were calculated as described in Materials and methods. The results are presented as the means ± S.E.M. of 4 independent experiments. Statistical significance is indicated (***P < 0.001, one-way ANOVA with Tukey’s post-hoc test). (**b**) The absorbance spectra of the same samples were measured in the range from 530 to 576 nm 10 min after the addition of rCytC. Reduced CytC shows a characteristic peak at A_550_. Inhibition of enzymic CCOX activity is indicated by the higher A_550_ peak height as compared to the positive control (MT). In case of all treatments, the CCOX activity was inhibited but to different extents. The spectrum is a representative result of one experiment in duplicates. The values represent the means ± S.E.M. for any given wavelength.

As shown, the neurotoxic sPLA_2_ interacts with CCOX in neuronal cells. It binds to CCOX subunit II, which is exposed to the intermembrane space (IMS) in mitochondria. This raised the question as to whether such binding has some influence on the enzymic activity of CCOX or not. To answer this question, mitochondria from PC12 cells were isolated and incubated at room temperature with Atx or other substances as defined under Materials and methods, followed by the addition of the CCOX substrate, reduced form of cytochrome c (rCytC). The reaction catalysed by CCOX is the oxidation of rCytC to CytC, which can readily be traced by measuring the absorption of the reaction mixture at 550 nm (A_550_), where rCytC has a distinctive absorption maximum but CytC does not. KCN, a specific inhibitor of CCOX activity, significantly reduced the rCytC oxidation rate by our mitochondrial preparation (Fig. 6a), confirming the involvement of CCOX in the process. The addition of 1 µM Atx to the suspension of mitochondria significantly reduced the rCytC oxidation rate relative to that in the absence of Atx (Fig. 6a). Interestingly, the inhibition of rCytC oxidation was apparently even more intense in the presence of Atx(D49S), the enzymically inactive mutant of Atx. This mutant was also able to inhibit the binding of ^125^I-Atx to R25 (Ivanušec *et al*., in preparation), suggesting that the inhibition of CCOX enzyme activity by sPLA_2_ is affected by the physical interaction of the two proteins. That Atx inhibits the activity of CCOX is further reflected in the fact that the relative rCytC oxidation rate was not reduced even more if the mitochondrial preparation was exposed to KCN and Atx or Atx(D49S) simultaneously (Fig. 6a). Differences in the CCOX inhibition potency between different substances are nicely presented also by comparing the heights of the rCytC-characteristic A_550_ peak of respective reaction mixtures 10 min past the start of the reaction (Fig. 6b). At this time the mitochondrial preparation (positive control) completely oxidized the added rCytC. Consistent with the influence on the rCytC oxidation rate, the inhibition of CCOX activity was most efficient with KCN, and the mixtures of KCN and Atx or Atx(D49S). Less efficient, but clear inhibition was achieved by Atx or Atx(D49S). The rCytC oxidation rates prior to normalization can be found in the Supplementary Fig. S1. Altogether, the results show that Atx and its enzymically inactive mutant significantly inhibit the activity of CCOX in mitochondria.

## Discussion

Although the presence of sPLA_2_ molecules inside cells has been reported^10,21,22^, their intracellular functions have remained practically unknown. Proteins containing 5 to 8 disulphide bridges would be expected to unfold rapidly in the reducing environment of the eukaryotic cytosol, with consequent loss of biological activity. In addition, the millimolar concentrations of the catalytic cofactor Ca^2+^ required to fully express the enzymic activity are, normally, not available inside the cytosol. However, *in vitro* experiments with Atx, the nose-horned viper venom β-ntx, and some mammalian sPLA_2_s have suggested the opposite ─ sPLA_2_s were well able to maintain both the structural integrity and the considerable enzymic activity in such an environment^23–26^. Considering these facts, the key results on the β-neurotoxic action of sPLA_2_s^3–6,27,28^ have led to the interpretation that this activity is, predominantly, the consequence of the intracellular action of these toxins^29^. β-neurotoxic snake venom sPLA_2_s have emerged as suitable tools for studying the intracellular pathophysiology of their mammalian counterparts, GIB and GIIA sPLA_2_s^1^. Their intracellular pathways are expected to be alike and they should share at least some of the intracellular interacting proteins (reviewed in^2^). Among the latter may well be R25, the first intracellular integral membrane sPLA_2_ receptor, which we have isolated and identified in this work.

Porcine cerebral cortex has been demonstrated to be an appropriate source in which to characterize neuronal receptors for Atx. While the neuronal M-type sPLA_2_ receptor^30^ and the soluble proteins, CaM, 14-3-3 protein and protein-disulphide-isomerase^31–33^, have been successfully identified as the Atx-binding proteins using this tissue, a membrane receptor of Atx with an apparent molecular mass of 25 kDa (R25), although the first to be detected^7^, persistently resisted purification and molecular identification. In this work, we were finally successful in this, due to some crucial improvements of the isolation procedure. To reduce the viscosity and ease the subsequent purification steps the membranes were extracted with 1.5% (m/v) Triton X-100, the lowest concentration of the detergent that still enabled effective solubilisation of R25 from the membranes^8^. For successful isolation of the receptor, development of an effective Atx-affinity chromatography protocol was essential. Optimal association between the toxin and the solubilized receptor was achieved at pH 8.2 in the presence of millimolar Ca^2+^, under conditions described as optimal also for the binding of ^125^I-Atx to R25 in membranes^7^. These conditions also enabled maximum facilitation of the binding of R25 from the detergent extract of the membranes to the Atx immobilized on the chromatographic support and, also, during the thorough washing of the Atx-affinity media in the next step of the purification procedure. Thirdly, removal of the non-specifically adsorbed proteins was also enhanced by washing the P2d membranes with a buffer containing a high concentration of NaCl prior to protein extraction and chromatography. Previous studies offered two possibilities for specific elution of the bound material from the Atx-affinity support ─ elution by lowering the pH of the mobile phase or by removal of Ca^2+^ ions from the media. As demonstrated, the affinity between R25 and Atx was maximal in the pH range 7.4 to 9 but dropped to zero below pH 5.5^7^. Similarly, removal of divalent ions from the system completely abolished the ability of Atx to bind R25^30^. Ca^2+^ ions are important in determining structural integrity, (and, hence, catalytic activity) of sPLA_2_s, and, consequently, interactions of these proteins with other molecules. Detergent-solubilized R25 proved prone to irreversible denaturation under acidic conditions, which prevented its detection in collected fractions by Atx-affinity labelling. On the other hand, the affinity between Atx and solubilized R25 depends reversibly on the concentration of Ca^2+^ ions. So, following the elution of R25 from the Atx-affinity support by removing Ca^2+^ ions with EGTA, the detection of R25 in collected fractions was again enabled by supplementing the fractions with an excess of Ca^2+^ (Fig. 1). A further and very important factor in purifying a biologically active R25 appeared to be the rate of execution of the Atx-affinity chromatography step. Cartridge-packed Atx-derivatized CIM disks on the FPLC system enabled the required speed, which was not achievable using classical affinity chromatography supports based on Affigel or CH-Sepharose.

Partial sequencing of the Atx-binding protein band with an apparent molecular mass of about 25 kDa suggested its identity as the subunit II of CCOX (Fig. 2b). The result that CCOX is indeed the receptor for Atx, the β-neurotoxic sPLA_2_ from the nose-horned viper venom, was confirmed by affinity-labelling of commercially available CCOX with two different Atx-derivatives, ^125^I-Atx and sulfo-SBED-Atx (Fig. 3). Considerable colocalization of signals for Atx and CCOX-II in PC12 cells (Fig. 5) and the fact that the binding of Atx to CCOX-II, like that to R25, is also Ca^2+^-dependent^7,30^, further support the conclusion that R25 and CCOX-II are the same protein. In the complex of a dimer of 13 different subunits constituting CCOX in the inner mitochondrial membrane, the subunit II is positioned to face the mitochondrial IMS^18^ in a manner appropriate to receiving Atx arriving from the cytosol.

Besides participating in thermogenesis, steroid synthesis and apoptosis, mitochondria are very important in the production of ATP. Oxidative phosphorylation begins with the entry of electrons into the chain of electron carriers, the respiratory chain. The mitochondrial respiratory chain consists of a series of sequentially acting electron carriers, most of which are integral proteins with prosthetic groups capable of accepting and donating either one or two electrons^34,35^. We have demonstrated here that Atx as well as its enzymically inactive mutant Atx(D49S) inhibit the activity of CCOX (termed also complex IV) of the respiratory chain. As evident from the experiments including KCN, a specific inhibitor of CCOX activity, the reduction of rCytC oxidation rate by the mitochondrial preparation was solely due to the inhibition of CCOX activity by Atx or Atx(D49S) (Fig. 6). By inhibiting CCOX function with the enzymically inactive mutant of Atx, we demonstrated that the phospholipase activity is not indispensable to observe this effect. This is consistent with the observation that neurotoxicity was exerted also by enzymically inactive mutant of β-ntx^36^. Clearly, just binding of such sPLA_2_ molecules to CCOX-II is enough to achieve the inhibition of the oxidation of rCytC. As subunit II is one of the three subunits (also I and III) critical for the functioning of CCOX^37^, this is not surprising. Interestingly, Atx(D49S) was shown to inhibit the oxidation of rCytC by mitochondrial preparation to a higher extent than Atx (Fig. 6). This could be due to the fact that in the experimental setup used, Atx damaged the outer mitochondrial membrane by hydrolysing it and thus exposing additional CCOX to the substrate, resulting in its more efficient oxidation. CCOX carries electrons from rCytC to molecular oxygen and pumps protons from the matrix into the IMS^38,39^. By hydrolysing phospholipids, Atx releases free fatty acids, known to be modulators of CCOX activity^40^ and uncouplers of oxidative phosphorylation^41^. Secreted PLA_2_ may therefore participate in tuning oxidative phosphorylation by inhibiting CCOX activity through formation of free fatty acids and/or by physical interaction with the CCOX. The latter way is probably more important under conditions that do not support the phospholipase activity at low micromolar Ca^2+^ and acidic pH. In addition, the association of GIIA sPLA_2_ with CCOX-II may also aid proper positioning of the enzyme in the organelle for its targeted enzymic action.

Energy depletion at the nerve ending, due to the specific effect on mitochondria, appears to be the major reason for neuro-muscular blockade in the case of Atx, the GIIA β-ntx^42^. It causes disruption of the coupling between nerve terminal depolarization and consequent neurotransmitter release. In the case of the GI β-ntxs, such as notexin, β-bungarotoxin, taipoxin and textilotoxin, a radical depletion of the number of synaptic vesicles in the nerve ending, which was not observed in the case of Atx^42^, is probably a more important cause of neuro-transmission failure^27^. In support of such an explanation of the observed pathological differences between these two types of β-ntxs, binding of ^125^I-Atx to R25, *i*.*e*. CCOX-II, was not inhibited by taipoxin or by β-bungarotoxin^7^.

The mitochondrion is one of the most exciting cellular compartments regarding the pathophysiology of sPLA_2_s. Endogenous GIIA sPLA_2_ has been detected in the mitochondrion under normal physiological conditions, under which it is apparently harmless to this organelle^10^. Apart from its role in neuritogenesis^11^, it has been speculated that this enzyme is also important for the fitness of the mitochondrion, due to its ability to hydrolyse oxidized fatty acid chains arising from its phospholipids such as cardiolipin, for assisting remodelling of mitochondrial membranes during fusion and fission of the organelle and during mitophagy^12,43^. Activation of the phospholipase activity in mitochondria is, on the other hand, connected, importantly, with excitotoxicity, oxidative stress and with the mitochondrial dysfunction due to induction of the mitochondrial permeability transition^12^. Many results support the idea that the increased activity of GIIA sPLA_2_ participates in the development of neurodegeneration, either following short-term ischemia or as the consequence of the long-term neurodegenerative diseases that occur when mitochondrial functions undergo substantial alterations that reduce their ATP-producing capability and direct neurons toward necrotic or apoptotic cell death^10^. Elevated activity of GIIA sPLA_2_ affects mitochondria in a way reminiscent of β-ntxs leading to neurodegeneration^15–17^. This occurs under conditions of reduced respiratory activity^10^ or of increased concentration of GIIA sPLA_2_ in the extracellular space provided by reactive astrocytes and activated microglia^16^. Whether the endogenous GIIA sPLA_2_ also binds to CCOX is not yet known. It is however known that its association with the inner mitochondrial membrane is dependent on Δ*Ψ*_m_, which is negative on the matrix side^10,44^. When Δ*Ψ*_m_ is dissipated, due for example to the absence of respiratory substrates, GIIA sPLA_2_ is released from the membrane and exits mitochondria. Both the endogenous GIIA sPLA_2_ and Atx are very basic proteins, so we may expect that Δ*Ψ*_m_ is a factor that also dictates the affinity between Atx and CCOX-II.

In conclusion, in this work the mitochondrial receptor for Atx, the β-neurotoxic snake venom sPLA_2_, has been characterized as the subunit II of the CCOX. This is the first intracellular integral membrane receptor for sPLA_2_ described so far and may provide a new and important detail to the understanding of β-neurotoxicity at the molecular level. Due to similarities between endogenous GIIA sPLA_2_, resident in mitochondria, and Atx, the knowledge gained in this study opens an important direction of study to advance understanding of the mitochondrial function and dysfunction of this mammalian enzyme, especially in the nervous system.

## Materials and Methods

### Preparation of mitochondrial membranes and extraction of their proteins

Porcine brain was purchased from a local slaughterhouse. Porcine cerebral cortex membrane fractions were isolated as described previously^8^. The mitochondrial fraction P2d (total protein concentration 3 mg/mL) was combined with 2 M NaCl in “Label Transfer Buffer” (LTB; 75 mM Hepes, pH 8.2, 150 mM NaCl, 2 mM CaCl_2_) at a 1 : 1 (v/v) ratio, mixed for 60 min at 4 °C and finally centrifuged at 14,000 × *g* for 60 min at 4 °C. The supernatant was discarded and the pellet re-suspended in LTB and centrifuged again at 14,000 × *g* for 60 min at 4 °C. The supernatant was discarded and the pellet re-suspended in LTB to a final total protein concentration of 3 mg/mL. Membrane proteins were then extracted for 1 h at 4 °C by gentle agitation of the suspension in LTB containing 1.5% (m/v) Triton X-100. The extracts were centrifuged for 60 min at 4 °C at 14,000 ×*g* and the supernatants collected.

### Isolation of Atx-binding proteins on Atx-affinity CIM disks

Atx-affinity Convective Interaction Media (CIM) disks (BIA Separations, Slovenia) were prepared as described^45^. Atx-binding proteins were isolated on an ÄKTA FPLC apparatus (Amersham Pharmacia Biotech, Sweden). The disks were first equilibrated in LTB containing 0.1% (m/v) Triton X-100 (LTBT). 2 mL of P2d detergent extract was then applied onto the disks at a flowrate of 0.02 mL/min. Unbound material was washed off with 30 mL of LTBT at 0.3 mL/min. Bound proteins were then eluted at the same flowrate with LTBT containing 0.5 mM EGTA. Eluted fractions were immediately supplemented with CaCl_2_ to a final concentration of 5 mM and affinity-labelled with ^125^I-Atx. Fractions containing Atx-binding proteins were concentrated and analysed by SDS-PAGE. Proteins were stained with colloidal silver^46^. R25-containing piece of gel was excised and processed for LC-MS and MS/MS analyses, as described below.

### LC-MS/MS protein identification

Mass spectrometry (MS) was performed as described^47^. Specifically, the LC-MS/MS system consisted of a Thermo Electron (USA) linear ion trap Fourier transform (LTQ FT) hybrid mass spectrometer and a Protana (Denmark) nanospray ion source interfaced to a self-packed 8 cm × 75 µm Phenomenex (USA) Jupiter 10 µm C18 reversed-phase capillary column. The R25-containing piece of gel was destained overnight, reduced with dithiothreitol, alkylated with iodoacetamide, and digested with Promega (USA) modified trypsin for 16 h. Peptides were extracted from the gel with 50% (v/v) acetonitrile/5% (v/v) formic acid. 25% of the peptide digest extract was injected and the peptides eluted from the column by a gradient of acetonitrile/0.1 M acetic acid at a flow rate of 0.25 µL/min. The nanospray ion source was operated at 2.8 kV. The digest was analyzed using a repeating cycle of 1 MS (100 K FT) followed by 10 MS/MS (FT-IT mode). The instrument used the following settings – 35 normalized collision energy, 3 isolation window, dynamic exclusion (repeat count 1, repeat duration 30 s, and exclusion duration 120 s). The data were analyzed by database searching using the Sequest search algorithm within Thermo Bioworks 3.0 (USA) against NCBI’s NR database. The search parameters were: parent tolerance 10 ppm, fragment tolerance 1.0 Da, carbamidomethyl Cys fixed, and oxidation Met variable. The resulting data were filtered by Sequest score function *Xcorr*: +1 > 1.5, + 2 > 1.8, + 3 > 2.2, and +4 > 2.5 to produce a master protein list with a low false discovery rate (FDR). Peptides for proteins of particular interest were examined manually for further validation.

### ^125^I-Atx affinity-labelling

To a detergent extract of porcine brain P2d mitochondrial membranes, diluted in LTB to 0.8% (m/v) Triton X-100, or to 200 nM purified bovine heart CCOX (Sigma-Aldrich, USA) in LTBT or LTBT in which Ca^2+^ was substituted with 2 mM EGTA, ^125^I-Atx was added to a final concentration of 10 nM. The reaction mixture was incubated at room temperature for 30 min. The control sample contained 200-fold molar excess over ^125^I-Atx of the unlabeled Atx or of enzymically inactive Atx(D49S) mutant (Ivanušec *et al*., in preparation). After the incubation, disuccinimidyl suberate (DSS; Pierce, USA) was added to 100 µM final concentration. After 5 min, the cross-linking reaction was stopped by adding the reducing SDS-PAGE loading buffer. Samples were analysed by SDS-PAGE and the gels dried and autoradiographed at −70 °C using Kodak X-Omat AR films^30^.

### Culturing of PC12 cells

Rat PC12 cells ATCC CRL-1721 (American Type Culture Collection, USA) were grown in 10-cm culture plates in F12K (Kaighn’s modification of Ham’s F-12) growth medium (Gibco, USA) containing 15% (v/v) horse serum, 2.5% (v/v) fetal bovine serum, 100 units/mL of penicillin and 100 μg/mL streptomycin at 37 °C and 5% (v/v) CO_2_. For immunofluorescence studies, the cells were plated on poly-*L*-Lys coated coverslips and grown on 6-well plates under the same conditions as described above.

### Detection of the specific 25 kDa Atx-binding protein in PC12 cells

The mitochondrial membranes-containing P2d fraction was isolated from PC12 cells^8^ and affinity-labelled with ^125^I-Atx as described^7^.

Sulfo-SBED-Atx was synthesized according to the published protocol^48^. PC12 cells were affinity-labelled using sulfo-SBED-Atx^20^. Intact PC12 cells, plated on 10 cm plates at 80 to 90% confluence, were treated with sulfo-SBED-Atx at a concentration of 100 nM for 1, 2, 5, 15, 30 and 60 min in HBSS in the dark at 37 °C under 5% (v/v) CO_2_. In a control experiment, the cells were incubated for 5 min without addition of sulfo-SBED-Atx. Subsequently, the cells were cooled on ice, exposed for 5 min to five 15 W 312 nm UV lamps from a distance of 5 cm and washed twice with HBSS. The cells were then immersed in 1.8 mL 1.5% (m/v) Triton X-100/HBSS for 15 min to extract the proteins. The extracts were centrifuged for 45 min at 14,000 × *g* and 4 °C. Supernatants were incubated with 375 μL monomeric avidin agarose beads for 1 h at 4 °C. The beads were washed thoroughly with HBSS containing 0.1% (m/v) Triton X-100 and the biotin containing proteins then eluted with 2 mM *D*-biotin/0.1% (m/v) Triton X-100/HBSS. Atx-binding proteins in the eluted fractions were immuno-precipitated as follows: 400 μL aliquots of the avidin-chromatography eluates were incubated with 100 μL protein G-Sepharose coupled with polyclonal anti-Atx antibodies. The samples were incubated overnight at 4 °C. The beads were then washed extensively and the proteins eluted by adding reducing SDS-PAGE loading buffer and heating at 95 °C for 5 min. The biotinylated proteins were detected as described below.

### Sulfo-SBED-Atx affinity-labelling of purified cytochrome c oxidase

Affinity-labelling was performed as described^48^. To 0.5 µM purified bovine heart CCOX (Sigma-Aldrich, USA) in LTBT, an equimolar concentration of sulfo-SBED-Atx was added in complete darkness. The control sample contained a 100-fold excess of native Atx over the labelled toxin. The samples were incubated for 30 min at room temperature in complete darkness with gentle agitation. The samples were then placed on ice and exposed to UV light at 312 nm (five 15 W light bulbs), followed by addition of reducing SDS-PAGE loading buffer. The biotinylated proteins were detected as described below.

### Detection of biotinylated proteins

The biotinylated samples were analysed by SDS-PAGE; the proteins were transferred onto a PVDF membrane and detected with streptavidin-horseradish peroxidase (SA-HRP; Pierce, USA) according to the manufacturer’s instructions. After incubation with SA-HRP, the membranes were washed 4 times for 15 min with TBST (50 mM Tris-HCl, pH 7.5, 150 mM NaCl, 0.1% (m/v) Tween-20) and the blots then developed using the Lumi-Light Plus ECL substrate (Roche, Switzerland), Kodak Biomax ECL films and Kodak developing solutions.

### Immuno-fluorescence study of colocalization on PC12 cells

Colocalization study on PC12 cells was performed as described previously^20^. PC12 cells on poly–*L*–Lys coated coverslips were incubated at 37 °C under 5% (v/v) CO_2_ in the presence of 100 nM ^546^Alexa–Atx for 1, 2, 5, 15, 30 and 60 min. After the incubation, the cells were washed 3 times with DPBS at room temperature, and then fixed for 25 min in 4% (m/v) paraformaldehyde in DPBS at room temperature. After fixing, the cells were washed 4 times (5 min each) with ice cold DPBS and then incubated at room temperature in a blocking solution containing 0.5% (m/v) fish skin gelatin and 10% (m/v) fetal bovine serum in DPBS for 20 min. The coverslips were then immersed in primary antibody solution (anti-CCOX-II monoclonal antibodies, diluted 1 : 200 in blocking solution) (Thermo Fisher Scientific, USA). After 60 min incubation at room temperature, the cells were rinsed 5 times (5 min each) in DPBS, then incubated for 60 min at room temperature in the secondary antibody solution (Alexa Fluor 488 goat anti-mouse, diluted 1 : 2,000 in blocking solution) (Thermo Fisher Scientific, USA), followed by washing 4 times (5 min each) in DPBS. Excess fluid was removed and the slides mounted using ProLong Gold antifade reagent (Thermo Fisher Scientific, USA) and left to cure. After 24 h at room temperature in the dark, the edges of the coverslips were sealed with nail polish. The fixing, blocking and all washing steps were performed with gentle shaking in the dark. To determine the signals threshold, experiments were performed without the addition of the fluorescent probes. The control experiment to assess the signal stemming from the residual free ^546^Alexa dye in the preparation of ^546^Alexa–Atx was accomplished in the same way as the signal threshold experiments only in the presence of 500 nM ^546^Alexa with the inactivated reactive group (N-hydroxysuccinimide group).

The prepared slides were analysed on an inverted confocal laser scanning microscope (Axio Observer Z1 LSM 710, ZEISS, Germany) with a Plan-Apochromat 63/1.40 oil objective. Fluorophores were excited sequentially using argon (488 nm) and helium-neon (543 nm) lasers and the emitted light was collected through SP 545 and LP 545 filters. Stacks of fluorescent images were acquired. The signal thresholds for the red and green channels were set at a value just above the highest signal of the control experiment without the addition of the respective fluorescent probe. The thresholds were between 10% and 20% of the maximum signal intensity. The mean degree of colocalization of the red and green signals of at least two sets of images for each time point was calculated using ZEISS ZEN software. Colocalization was presented as Manders’ coefficient^49^, *i*.*e*. the ratio of the summed intensities of pixels from the green channel, for which the intensity in the red channel is above threshold, to the total intensity in the green channel. Images of colocalization were displayed using the “Colocalization” tab in the ZEISS ZEN software.

### Measurement of CCOX activity in mitochondria isolated from PC12 cells

PC12 cells mitochondria were isolated using the Qproteome Mitochondria Isolation Kit (Qiagen, Netherlands) according to the manufacturer’s instructions. CCOX activity was measured using the Cytochrome c Oxidase Assay Kit (Sigma-Aldrich, USA) according to the manufacturer’s instructions. Briefly, isolated mitochondria in buffer A (10 mM Tris-HCl, pH 7.0, 120 mM KCl) (6.1 µg/mL total protein) were incubated for 30 min at room temperature in the presence of either 1 µM Atx, 1 µM Atx(D49S) (enzymically inactive mutant of Atx), 0.5 mM KCN (specific inhibitor of CCOX), 1 µM Atx with 0.5 mM KCN, or 1 µM Atx(D49S) with 0.5 mM KCN. The positive control contained only mitochondria in buffer A, while the negative control only buffer A. Following incubation, the solution containing rCytC was added to the final concentration of 22 µM and the decrease in absorbance at 550 nm of the reaction mixture was recorded on a microplate reader (Tecan, Austria) for 10 min, the time in which rCytC in the positive control was completely oxidized. Then, immediately, the absorption spectra of the reaction mixture from 530 to 576 nm was measured on the same instrument. To determine the relative rate of rCytC oxidation in specified conditions, the average slope of the function y(t) = log (A_550_(t) − A_550_(600 s))^50^ in the first 300 s (linear part of the function) was calculated, the slope of the negative control subtracted and the obtained result normalized to the positive control. A_550_(600 s) was obtained in the positive control at time 600 s, when rCytC was completely oxidized. Statistical analysis was performed using Prism 7.0 (GraphPad Software, USA). The relative rCytC oxidation rates are presented as means ±S.E.M. (standard error of mean) of the 4 independent experiments. Statistical significance was determined using one-way ANOVA, followed by Tukey’s post hoc test. P values lower than 0.05 were considered statistically significant.

## Electronic supplementary material

Supplementary Figure S1

## Data Availability

The datasets generated during and/or analysed during the current study are available from the corresponding author on reasonable request.
