# Supplementary material for: The neurotoxic secreted phospholipase A2 from the Vipera a. ammodytes venom targets cytochrome c oxidase in neuronal mitochondria
Source: Sci Rep. 2019 Jan 22;9:283. doi: 10.1038/s41598-018-36461-6 (PMC6342964; doi:10.1038/s41598-018-36461-6)
Supplement: Supplementary file 1 — Supplementary Figure S1 [file 41598_2018_36461_MOESM1_ESM.pdf]

The neurotoxic secreted phospholipase A_2 from the *Vipera a. ammodytes* venom targets cytochrome c oxidase in neuronal mitochondria

Jernej Šribar[1], Lidija Kovačič[1], Jernej Oberčkal[1], Adrijan Ivanušec[1,2], Toni Petan[1], Jay W. Fox[3] and Igor Križaj[1*]

[1] Department of Molecular and Biomedical Sciences, Jožef Stefan Institute, Jamova 39, 1000 Ljubljana, Slovenia

[2] Faculty of Medicine, University of Ljubljana, Vrazov trg 2, 1000 Ljubljana, Slovenia

[3] Department of Microbiology, Immunology and Cancer Biology, University of Virginia School of Medicine, Charlottesville, Virginia 22908, USA

*igor.krizaj@ijs.si

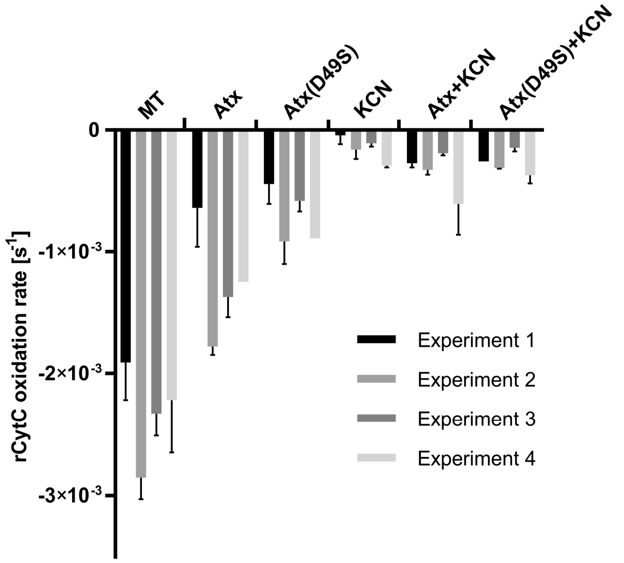

Figure S1. rCytC oxidation rates by isolated PC12 mitochondria in different conditions. Mitochondria were isolated from PC12 cells and incubated in either the absence (MT) or the presence of 1 μM Atx, 1 μM Atx(D49S), 0.5 mM KCN, 1 μM Atx with 0.5 mM KCN or 1 μM Atx(D49S) with 0.5 mM KCN. After the addition of the CCOX substrate, rCytC, the change in absorbance at 550 nm (A_{550}) was measured. The rCytC oxidation rates were calculated as described in Materials and methods, omitting the normalization to the positive control. The results are presented as the means ± S.E.M. of two replicates for each of the 4 independent experiments.
